# Associations between lifetime classic psychedelic use and cardiometabolic diseases

**DOI:** 10.1038/s41598-021-93787-4

**Published:** 2021-07-13

**Authors:** Otto Simonsson, Walter Osika, Robin Carhart-Harris, Peter S. Hendricks

**Affiliations:** 1 Department of Sociology, University of Oxford, Oxford, UK; 2 Department of Clinical Neuroscience, Center for Psychiatry Research, Karolinska Institute, Solna, Sweden; 3 Department of Neurobiology, Care Sciences and Society, Center for Social Sustainability, Karolinska Institute, Solna, Sweden; 4 Northern Stockholm Psychiatry, Stockholm Health Care Services, Region Stockholm, Stockholm, Sweden; 5 Centre for Psychedelic Research, Imperial College London, London, UK; 6 Department of Health Behavior, University of Alabama at Birmingham, Birmingham, UK

**Keywords:** Pharmacology, Neuroscience, Psychology, Cardiology, Diseases, Health care, Risk factors

## Abstract

The objective of the current study was to investigate the associations between lifetime classic psychedelic use and cardiometabolic diseases. Using data from the National Survey on Drug Use and Health (2005–2014), the present study examined the associations between lifetime classic psychedelic use and two types of cardiometabolic disease: heart disease and diabetes. Respondents who reported having tried a classic psychedelic at least once in their lifetime had lower odds of heart disease in the past year (adjusted odds ratio (aOR) = 0.77 (0.65–0.92), p = .006) and lower odds of diabetes in the past year (adjusted odds ratio (aOR) = 0.88 (0.78–0.99), p = .036). Classic psychedelic use might be beneficial for cardiometabolic health, but more research is needed to investigate potential causal pathways of classic psychedelics on cardiometabolic diseases.

## Introduction

Cardiometabolic diseases such as heart disease and diabetes are leading contributors to the global burden of disease^1^. While pharmacological treatment, intensive lifestyle modification, or both can delay or reverse the development of cardiometabolic diseases^2–5^, no study has thus far investigated the long-term cardiometabolic effects of classic psychedelics, which could potentially be administered both as a pharmacological treatment and as part of a program to facilitate healthy lifestyle changes.

The term classic psychedelics broadly refers to psychoactive substances known to act as agonists primarily at serotonin 2A receptors^6^, which are often categorized into three main classes: tryptamines, lysergamides, and phenethylamines^7^. Most notably, tryptamines include N,N-dimethyltryptamine (DMT), the DMT-containing admixture ayahuasca, and psilocybin; lysergic acid diethylamide (LSD) comprises the lysergamide class; and phenethylamines include mescaline and the mescaline-containing cacti peyote and San Pedro^8^. The evidence to date suggests that classic psychedelics have a good risk profile and can be effective in the treatment of several mental health conditions^6,9^, but recent research indicates that classic psychedelics may also have beneficial effects for a range of physical illnesses, including cardiometabolic diseases such as heart disease and diabetes^10,11^.

There are several mechanisms through which classic psychedelics might influence cardiometabolic health. First, research suggests that classic psychedelics may facilitate healthy lifestyle changes associated with a beneficial impact on cardiometabolic risk factors (e.g., diet, alcohol and tobacco consumption, and exercise)^11^. Second, classic psychedelics administered in a safe and supportive setting have been shown to improve mental health conditions associated with cardiometabolic diseases^12–16^. Third, classic psychedelics have anti-inflammatory and immunomodulatory properties of importance for both mental and cardiometabolic health^17–20^. Fourth, classic psychedelics have high affinity to serotonin receptor subtypes associated with cardiometabolic diseases (e.g., serotonin 2A and 2C receptors)^17,21^. In sum, classic psychedelics could have both direct and indirect effects that lead to better cardiometabolic health.

Previous research has found associations between lifetime classic psychedelic use and lower odds of being overweight or obese as well as lower odds of having hypertension in the past year^22,23^, which are risk factors of cardiometabolic disease. Using pooled data from the National Survey on Drug Use and Health (2005–2014), the present study therefore sought to investigate the associations between lifetime classic psychedelic use and two types of cardiometabolic disease: heart disease and diabetes. We hypothesized that lifetime classic psychedelic use would be associated with lower odds of heart disease in the past year as well as lower odds of diabetes in the past year.

## Results

Table 1 displays the percentage of respondents reporting heart disease or diabetes in the past year. As seen in the table, the prevalence of heart disease or diabetes in the past year among respondents who had ever used a classic psychedelic was approximately 51% and 52%, respectively, of that among respondents who had never used a classic psychedelic. Notably, the prevalence of heart disease or diabetes in the past year among respondents who had ever used a tryptamine (DMT, ayahuasca, or psilocybin) was approximately 45% and 41%, respectively, of that among respondents who had never used a tryptamine. It is noted, however, that these relationships do not control for the range of potential confounding factors.

**Table 1:** Percentage of respondents with heart disease or diabetes in the past year.

Lifetime classic psychedelic use	Heart disease in the past year	
	Yes	No
Yes	658 (2.29%)	54,077 (97.71%)
No	6,495 (4.49%)	314,977 (95.51%)
Lifetime tryptamine use	Yes	No
Yes	383 (1.95%)	39,683 (98.05%)
No	6,770 (4.41%)	329,371 (95.59%)
Lifetime LSD use	Yes	No
Yes	529 (2.50%)	36, 836 (97.50%)
No	6,624 (4.38%)	332,218 (95.62%)
Lifetime phenethylamine use	Yes	No
Yes	303 (3.59%)	13,007 (96.41%)
No	6,850 (4.22%)	356,047 (95.78%)
Lifetime classic psychedelic use	Diabetes in the past year	
	Yes	No
Yes	1,322 (3.95%)	53,400 (96.05%)
No	12,913 (7.69%)	308,532 (92.31%)
Lifetime tryptamine use	Yes	No
Yes	722 (3.06%)	39,336 (96.94%)
No	13,513 (7.59%)	322,596 (92.41%)
Lifetime LSD use	Yes	No
Yes	1,013 (4.13%)	36,341 (95.87%)
No	13,222 (7.53%)	325,591 (92.47%)
Lifetime phenethylamine use	Yes	No
Yes	546 (5.72%)	12,758 (94.28%)
No	13,689 (7.25%)	349,174 (92.75%)

The number of observations with heart disease in the past year was 376,207; the number of observations with diabetes in the past year was 376,167. The unweighted number of respondents are presented in each cell and the weighted percentage of respondents are presented within parentheses.

Table 2 presents results from the regressions on the associations between lifetime classic psychedelic use and heart disease in the past year as well as diabetes in the past year. As illustrated below, lifetime classic psychedelic use was uniquely associated with a 23% lower odds of heart disease in the past year and a 12% lower odds of diabetes in the past year. Among the three main classes of classic psychedelics, neither lifetime tryptamine use, lifetime LSD use, nor lifetime phenethylamine use were uniquely associated with heart disease or diabetes in the past year when simultaneously entered into the regression models, though the association between lifetime tryptamine use and diabetes in the past year approached conventional levels of significance.

**Table 2:** Lifetime classic psychedelic use and cardiometabolic diseases.

Variable	aOR (95% CI)	*p* value
**Heart disease in the past year**		
Model 1		
Lifetime classic psychedelic use	0.77 (0.65–0.92)	.006
Model 2		
Lifetime tryptamine use	0.85 (0.69–1.06)	.152
Lifetime LSD use	0.88 (0.73–1.07)	.199
Lifetime phenethylamine use	0.92 (0.75–1.13)	.402
**Diabetes in the past year**		
Model 1		
Lifetime classic psychedelic use	0.88 (0.78–0.99)	.036
Model 2		
Lifetime tryptamine use	0.86 (0.74–1.00)	.055
Lifetime LSD use	0.92 (0.80–1.06)	.236
Lifetime phenethylamine use	1.01 (0.86–1.19)	.891

The number of observations in the models with heart disease in the past year as dependent variable was 375,473; the number of observations in the models with diabetes in the past year as dependent variable was 375,434; *aOR* adjusted Odds Ratio, *CI* confidence interval. Odds ratios were adjusted for age, sex, ethnoracial identity, educational attainment, annual household income, marital status, self-reported engagement in risky behavior, lifetime use of cocaine, other stimulants, sedatives, tranquilizers, heroin, pain relievers, marijuana, phencyclidine (PCP), 3,4-methylenedioxymethamphetamine (MDMA/ecstasy), inhalants, smokeless tobacco, pipe tobacco, cigar, and cigarettes daily, and age of first alcohol use; see Supplementary Table S1 for additional analysis.

## Discussion

The results of this national survey-based study showed that lifetime classic psychedelic use was associated with both lower odds of heart disease in the past year and lower odds of diabetes in the past year, which indicates that classic psychedelic use might be beneficial for cardiometabolic health. The findings are novel and build on previous findings on the associations between lifetime classic psychedelic use and various markers of physical health^22–24^, but there are several limitations inherent in the study design that merit consideration. First, the cross-sectional design used in the present study limits causal inference. The regression models controlled for several potential confounders, but the associations could have been affected by latent variables that were not included in the dataset and could not be controlled for (e.g., a common factor that predisposes respondents to classic psychedelic use might also predispose them to salubrious lifestyle behaviors associated with cardiometabolic health). Second, there was no information in the dataset on the context of classic psychedelic use, dose used, or frequency of use. The analysis could therefore not evaluate context, dose, or frequency-specific associations. Third, the term “heart disease” covers a wide range of conditions and the term “diabetes” can refer to several metabolic disorders, including type 1 and type 2 diabetes. It is therefore possible that associations might vary across types of heart disease and diabetes.

There has been extensive research during the last decades on prevention and treatment of cardiometabolic diseases, including several comprehensive interventions designed to reduce lifestyle risk factors. Yet the potential long-term effects of classic psychedelic use on cardiometabolic health remains largely unknown. The findings in the present study reveal associations between lifetime classic psychedelic use and lower odds of heart disease in the past year as well as lower odds of diabetes in the past year. It demonstrates the need for further research to investigate potential causal pathways of classic psychedelics on cardiometabolic health (i.e., lifestyle changes, mental health benefits, anti-inflammatory and immunomodulatory characteristics, and affinity to specific serotonin receptor subtypes).

## Methods

### Data and population

The National Survey on Drug Use and Health (NSDUH) is an annual survey designed to measure the prevalence of substance use and mental health issues in the United States. The present study used pooled data from NSDUH survey years 2005 to 2014, which were the only survey years with items on heart disease and diabetes in the past year. While previous research has investigated the association between lifetime classic psychedelic use and having a heart condition and/or cancer in the past year (composite measure; p = 0.09)^23^, this study examined the unique associations with heart disease and diabetes in the past year. The NSDUH public-use data files are available on their homepage: https://www.datafiles.samhsa.gov/study-series/national-survey-drug-use-and-health-nsduh-nid13517.

### Variables

The dependent variables were: (1) having been told to have heart disease in the past year and (2) having been told to have diabetes in the past year. Both dependent variables derived from the following question:

### Which, if any, of these conditions did a doctor or other medical professional tell you that you had in the past 12 months?

Consistent with prior research^25^, the independent variable was lifetime classic psychedelic use. Respondents reporting that they had ever, even once, used DMT, ayahuasca, LSD, mescaline, peyote or San Pedro, or psilocybin were coded as positive for lifetime classic psychedelic use, whereas those indicating that they had never used any of these substances were coded as negative.

The control variables were age in years (18–25, 26–34, 35–49, 50–64, 65 or older); sex (male or female); marital status (married, divorced/separated, widowed, or never married); ethnoracial identity (non-Hispanic White, non-Hispanic African American, non-Hispanic Native American/Alaska Native, non-Hispanic Native Hawaiian/Pacific Islander, non-Hispanic Asian, non-Hispanic more than one race, or Hispanic); annual household income (less than US$20,000, US$20,000–49,999, US$50,000–74,999, or US$75,000 or more); educational attainment (fifth grade or less, sixth grade, seventh grade, eight grade, ninth grade, tenth grade, eleventh grade, twelfth grade, Freshman/13th year, Sophomore/14th year or Junior/15th, Senior/16th year or Grad/Prof School); self-reported engagement in risky behavior (never, seldom, sometimes, or always); lifetime cocaine use; lifetime marijuana use; lifetime 3,4-methylenedioxymethamphetamine (MDMA/ecstasy) use; lifetime phencyclidine (PCP) use; lifetime inhalants use; lifetime other stimulants use; lifetime sedatives use; lifetime pain relievers use; lifetime smokeless tobacco use; lifetime pipe tobacco use; lifetime cigar use; lifetime daily cigarette use; and age of first alcohol use (less than 13 years of age [Preteen], 13–19 years of age [Teen], more than 19 years of age [Adult], or never used). The control variables were coded as separate covariates and were the same as those used in a recent study analyzing the same NSDUH survey years^22^.

### Statistical analyses

The present study first used descriptive statistics to present an overview of the zero-order relationships of lifetime psychedelic use and subcategories of lifetime use of tryptamines (DMT, ayahuasca, or psilocybin), LSD, and phenethylamines (mescaline, peyote, or San Pedro) with both heart disease in the past year and diabetes in the past year (Table 1). These zero-order relationships were then interrogated further with logistic regression, which was used to calculate adjusted odds ratios with 95 percent confidence intervals and examine the unique associations between lifetime classic psychedelic use and cardiometabolic diseases while adjusting for the control variables listed above (Table 2). The analyses used weights provided by the NSDUH. “Bad Data”, “Don’t Know”, “Refused”, “Blank” were coded as missing values. The analyses were conducted using Stata version 17^26^.

### Ethical approval

The current study was a secondary analysis of publicly available data files and was exempt from review by the Research Ethics Committee of the Department of Sociology (DREC) at the University of Oxford.

## Supplementary Information

Supplementary Information.

## References

[1] Roth GA (2018). Global, regional, and national age-sex-specific mortality for 282 causes of death in 195 countries and territories, 1980–2017: A systematic analysis for the global burden of disease study 2017. Lancet.

[2] Arnett DK (2019). 2019 ACC/AHA guideline on the primary prevention of cardiovascular disease: a report of the American College of Cardiology/American Heart Association Task Force on Clinical Practice Guidelines. J. Am. Coll. Cardiol..

[3] Artinian NT (2010). Interventions to promote physical activity and dietary lifestyle changes for cardiovascular risk factor reduction in adults: a scientific statement from the American Heart Association. Circulation.

[4] Chatterjee S, Khunti K, Davies MJ (2017). Type 2 diabetes. Lancet.

[5] Tuomilehto J (2001). Prevention of type 2 diabetes mellitus by changes in lifestyle among subjects with impaired glucose tolerance. N. Engl. J. Med..

[6] Nutt D, Carhart-Harris R (2021). The current status of psychedelics in psychiatry. JAMA Psychiat..

[7] Szabo A (2015). Psychedelics and immunomodulation: Novel approaches and therapeutic opportunities. Front. Immunol..

[8] Sexton JD, Crawford MS, Sweat NW, Varley A, Green EE, Hendricks PS (2019). Prevalence and epidemiological associates of novel psychedelic use in the United States adult population. J. Psychopharmacol..

[9] Nutt DJ, King LA, Phillips LD (2010). Drug harms in the UK: A multicriteria decision analysis. Lancet.

[10] Frecska E, Bokor P, Winkelman M (2016). The therapeutic potentials of ayahuasca: Possible effects against various diseases of civilization. Front. Pharmacol..

[11] Teixeira, P. J. et al. Psychedelics and health behaviour change. *J. Psychopharmacol.* 02698811211008554 (2021). 10.1177/02698811211008554 PMC8801670 34053342

[12] Carhart-Harris R (2021). Trial of psilocybin versus escitalopram for depression. N. Engl. J. Med..

[13] Chaddha A, Robinson EA, Kline-Rogers E, Alexandris-Souphis T, Rubenfire M (2016). Mental health and cardiovascular disease. Am. J. Med..

[14] Davis AK (2021). Effects of psilocybin-assisted therapy on major depressive disorder: A randomized clinical trial. JAMA Psychiat..

[15] Luoma JB, Chwyl C, Bathje GJ, Davis AK, Lancelotta R (2020). A Meta-analysis of placebo-controlled trials of psychedelic-assisted therapy. J. Psychoactive Drugs.

[16] Sartorius N (2018). Depression and diabetes. Dialogues Clin. Neurosci..

[17] Nichols, C. D. Serotonin 5-HT2A receptor function as a contributing factor to both neuropsychiatric and cardiovascular diseases. *Cardiovasc. Psychiatry Neurol.* (2009). 10.1155/2009/475108 PMC2790184 20029624

[18] Flanagan TW, Nichols CD (2018). Psychedelics as anti-inflammatory agents. Int. Rev. Psychiatry.

[19] Furman D (2019). Chronic inflammation in the etiology of disease across the life span. Nat. Med..

[20] Thompson, C. & Szabo, A. Psychedelics as a novel approach to treating autoimmune conditions. *Immunol. Lett. ***288**, 45–54 (2020). 10.1016/j.imlet.2020.10.001 33035575

[21] Zhou L (2007). Serotonin 2C receptor agonists improve type 2 diabetes via melanocortin-4 receptor signaling pathways. Cell Metab..

[22] Simonsson O, Hendricks PS, Carhart-Harris R, Kettner H, Osika W (2021). Association Between Lifetime Classic Psychedelic Use and Hypertension in the Past Year. Hypertension.

[23] Simonsson O, Sexton JD, Hendricks PS (2021). Associations between lifetime classic psychedelic use and markers of physical health. J. Psychopharmacol..

[24] Ona G (2019). Ayahuasca and public health: Health status, psychosocial well-being, lifestyle, and coping strategies in a large sample of ritual ayahuasca users. J. Psychoactive Drugs.

[25] Hendricks PS (2018). The relationships of classic psychedelic use with criminal behavior in the United States adult population. J. Psychopharmacol..

[26] StataCorp (2021). Stata statistical software: Release 17.

