# Supplementary material for: Associations between lifetime classic psychedelic use and cardiometabolic diseases
Source: Sci Rep. 2021 Jul 13;11:14427. doi: 10.1038/s41598-021-93787-4 (PMC8277805; doi:10.1038/s41598-021-93787-4)
Supplement: Supplementary file 1 — Supplementary Information. [file 41598_2021_93787_MOESM1_ESM.docx]

**Associations between lifetime classic psychedelic use and cardiometabolic diseases**

Authors: Otto Simonsson^1, 2,^ *, Walter Osika^2, 3, 4^, Robin Carhart-Harris^5^, Peter S. Hendricks^6^

^1^Department of Sociology, University of Oxford

^2^Center for Psychiatry Research, Department of Clinical Neuroscience, Karolinska Institute

^3^Center for Social Sustainability, Department of Neurobiology, Care Sciences and Society, Karolinska Institute

^4^Northern Stockholm Psychiatry, Stockholm Health Care Services, Region Stockholm

^5^Centre for Psychedelic Research, Imperial College London, UK

^6^Department of Health Behavior, University of Alabama at Birmingham

*otto.simonsson@trinity.ox.ac.uk

**Supplementary Information**

In response to a reviewer request that we evaluate whether the findings were selective for classic psychedelics, we also tested the association between cardiometabolic diseases and lifetime use of marijuana, a substance with some overlap in pharmacology and phenomenology with classic psychedelics ^[1], [2]^. These analyses were not part of our a priori analytic plan. The results showed that there was no significant associations between lifetime marijuana use and heart disease in the past year or lifetime marijuana use and diabetes in the past year (see Supplementary Table S1).

| Supplementary Table S1. Lifetime use of marijuana and cardiometabolic diseases | | | |
| --- | --- | --- | --- |
| Variable | aOR (95% CI) | *p* value |  |
|  | | |  |
| Heart disease in the past year | | | |
| Lifetime marijuana use | 0.93 (0.85-1.02) | .142 |  |
|  |  |  |  |
| Diabetes in the past year | | | |
| Lifetime marijuana use | 0.99 (0.92-1.07) | .887 |  |
|  |  |  |  |
| The number of observations in the models with heart disease as dependent variable was 375,473; the number of observations in the models with diabetes as dependent variable was 375,434; aOR: adjusted Odds Ratio; CI: confidence interval. Odds ratios were adjusted for age, sex, ethnoracial identity, educational attainment, annual household income, marital status, self-reported engagement in risky behavior, lifetime use of classic psychedelics, cocaine, other stimulants, sedatives, tranquilizers, heroin, pain relievers, phencyclidine (PCP), 3,4-methylenedioxymethamphetamine (MDMA/ecstasy), inhalants, smokeless tobacco, pipe tobacco, cigar, and cigarettes daily, and age of first alcohol use. | | | |

**References**

1. Ibarra-Lecue, I., Mollinedo-Gajate, I., Meana, J. J., Callado, L. F., Diez-Alarcia, R., & Urigüen, L. Chronic cannabis promotes pro-hallucinogenic signaling of 5-HT2A receptors through Akt/mTOR pathway. *Neuropsychopharmacology*, 43(10), 2028-2035 (2018).
2. Franklin, J. M., & Carrasco, G. A. Cannabinoid-induced enhanced interaction and protein levels of serotonin 5-HT2A and dopamine D2 receptors in rat prefrontal cortex. *Journal of Psychopharmacology*, 26(10), 1333-1347 (2012).
